# Gas-phase UV absorption spectra and OH-oxidation kinetics of 1*H*-1,2,3-triazole and pyrazole†

**DOI:** 10.1039/c9ra04235k

**Published:** 2019-08-30

**Authors:** Brahim Samir, Carmen Kalalian, Estelle Roth, Rachid Salghi, Abdelkhaleq Chakir

**Affiliations:** Groupe de Spectrométrie Moléculaire et Atmosphérique GSMA, UMR CNRS 7331, Université de Reims Moulin de la Housse B.P. 1039 51687 Reims Cedex 2 France abdel.chakir@univ-reims.fr; Laboratory of Environmental Engineering and Biotechnology, ENSA, University Ibn Zohr PO Box 1136 80000 Agadir Morocco

## Abstract

In this work, we report the gas phase UV absorption spectra and the kinetics of the OH-oxidation of 1*H*-1,2,3-triazole and pyrazole. UV spectra were determined between 200 and 250 nm, at 350 ± 2 K and at pressures between 0.09 and 0.3 Torr. The reported maximal UV absorption cross sections are (cm^2^ per molecule): *σ*_206 nm, 1*H*–1*H*-1,2,3-triazole_ = 2.04 × 10^−18^ and *σ*_203 nm, pyrazole_ = 5.44 × 10^−18^. The very low absorption capacity of these compounds beyond 240 nm indicates that their atmospheric photodissociation is negligible. The OH-oxidation of these species was performed in an atmospheric simulation chamber coupled to an FTIR spectrometer and to a GC/MS over the temperature range 298–357 K and at atmospheric pressure. Experiments were conducted in relative mode using benzaldehyde, *trans*-2-hexenal and heptane as references. The obtained rate constants at 298 K were (×10^−11^ cm^3^ per molecule per s): *k*(OH + 1*H*-1,2,3-triazole) = 2.16 ± 0.41; *k*(OH + pyrazole) = 2.94 ± 0.42. These results were compared to those available in the literature and discussed in terms of structure-reactivity and temperature dependency. Their tropospheric lifetimes with respect to reaction with OH radicals were then estimated.

## Abstract 2

In this work, we report the gas phase UV absorption spectra and the kinetics of the OH-oxidation of 1*H*-1,2,3-triazole and pyrazole.

## Graphical abstract

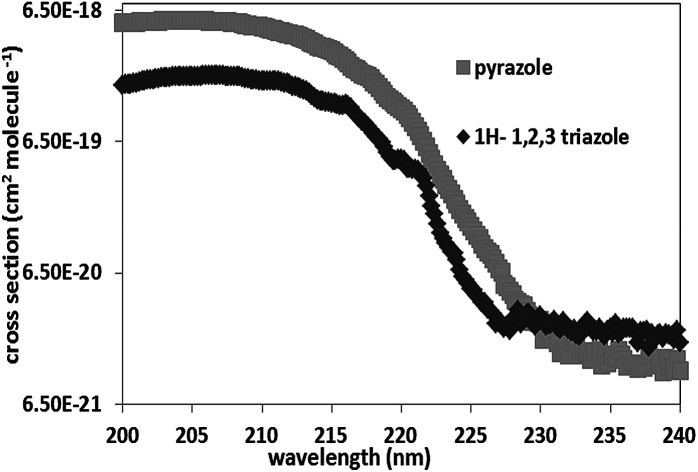

## 1. Introduction

Five-membered nitrogen heterocycles with a triazole or pyrazole moiety play an important role in organic chemistry: they are present in many organic species including natural, medicinal, and agricultural products.^1–3^ Most of pharmaceuticals are products that mimic natural products with biological activity and are heterocyclic compounds with a triazole or/and pyrazole ring systems. Indeed the Comprehensive Medicinal Chemistry (CMC) database lists more than 67% of compounds contain heterocyclic rings as pesticides (insecticides, fungicides) as well as antiviral and antibacterial agents.^4–7^ Furthermore, these compounds are also used as in several industrial applications such as in polymers,^8^ corrosion inhibitors,^3,9^ ligands for transition metals,^10,11^ cosmetic colorings,^2,8,12^ solvents, antioxidants and UV stabilizers.^8,12,13^ Hence, the usage of such nitrogen heterocycles species leads to their release into the atmosphere. Indeed, atmospheric measurements conducted by Jakob *et al.* and Teich *et al.*^14,15^ detected the presence of five-membered nitrogen heterocycles in aerosols. Among these heterocyclic compounds, imidazole derivatives were observed at concentrations varying from 0.2 to 14 ng m^−3^.^15^ Once in the atmosphere they can be oxidized through different chemical reactions with the atmospheric photo-oxidants (OH, Cl and NO_3_ radicals, and ozone). Unfortunately, gas phase kinetic studies of the atmospheric reactivity of these compounds with respect to the various photo-oxidants are scarce due to difficulties of handling these species with relatively low vapor pressures. Previous studies only focused on the atmospheric reactivity of pyrrole.^16–18^ These studies have shown that this compound is non-persistent with an atmospheric lifetime of the order of few hours. Its reaction with OH radicals proceeds *via* hydrogen abstraction from the NH bond along with the addition of the OH radical to the double bonds. The addition reaction is probably the most dominant channel.^16–18^ Moreover, temperature studies showed that this reaction exhibits a negative temperature dependence.^16–18^ Pyrazole and 1*H*-1,2,3-triazole UV spectra have been measured in two different studies to investigate their electronic structure and valence spectroscopy using synchrotron as a radiation source.^19,20^ Walker *et al.*^19^ determined the UV spectrum of pyrazole in the spectral range 110–250 nm at ambient temperature. However, the main objective of this study^19^ was the determination of the bands positions.

Palmer *et al.*^20^ measured the vacuum ultraviolet photoabsorption spectrum of 1*H*-1,2,3-triazole in the spectral range of 115–245 nm at 35 °C. The obtained measurements were analyzed using the comparison of the UV valence photoelectron ionizations and the results of *ab initio* configuration interaction (CI) calculations. However, the reported cross sections were very noisy beyond 220 nm. Thus, more accurate UV spectra of these species are required. To understand the atmospheric reactivity and to enrich kinetic and spectroscopic databases regarding these species further laboratory measurements are needed. For this purpose, in this work the UV spectra of the simplest five-membered nitrogen heterocycles, namely 1*H*-1,2,3-triazole and pyrazole alongside their kinetic degradation by OH radicals are investigated. This work provides the first kinetic data for the reactions of 1*H*-1,2,3-triazole and pyrazole with OH as a function of temperature. The obtained spectra are compared with previous studies, and their atmospheric lifetimes with respect to OH radicals are estimated. The chemical structures of the investigated compounds are given below.
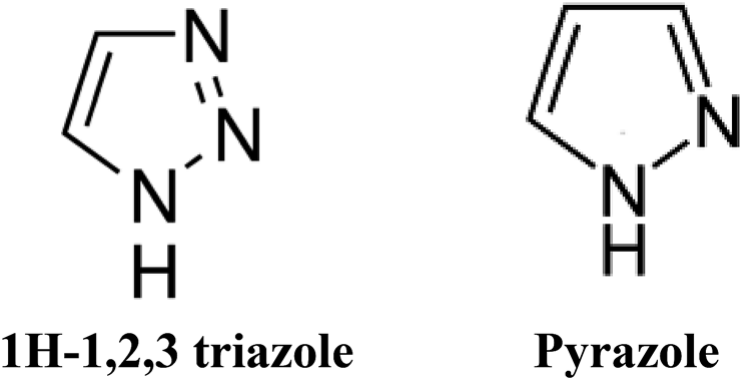


## 2. Materials and methods

### 2.1. UV-spectra

The experimental setup and methodology used in this paper were detailed in previous publications.^21,22^ In brief, the light source consisted of a 30 W deuterium lamp providing a continuum extending from 200 to 400 nm. A lens was used to focus the light into a calibrated monochromator (Horiba Jobin-Yvon iHR 320 – focal length 320 nm) equipped with a 1800 line mm^−1^ grating (dispersion: 2.35 nm mm^−1^; resolution: 0.06 nm). At the exit of the monochromator, light was focused towards the absorption cell (double-jacket Pyrex reactor, 100 cm in length and 2 cm in diameter, equipped with quartz windows). A photomultiplier (PM Hamamatsu R955) was used to detect the light signal. A circulation of heated water between the two walls of the reactor, using a Lauda Ultra R410 type thermostat, allows to regulate the temperature. Two platinum resistance temperature sensors (Pt 100-DIN 43760) were used to measure the temperature inside the cell. The pressure in the chamber was measured by a 0–10 Torr MKS Baratron capacitance manometers. The absorption cross-sections were calculated according to Beer Lambert's law. Thus, the cross-section *σ*(*λ*) at wavelength (*λ*) in (cm^2^ per molecule) is given by:I
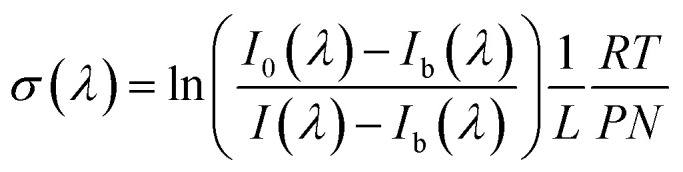
where *L* is the optical path (100 cm), *R* the perfect gas constant, *T* the temperature, *P* the pressure and *N* the Avogadro's constant. *I*_b_(*λ*) is the residual background noise, *I*_0_(*λ*) the intensity of the issued radiation measured under vacuum in the absence of absorbing species and *I*(*λ*) the transmitted intensity measured at each wavelength in the presence of the analyte.

Experiments were carried out under static regime, in the spectral range 200–300 nm, at temperature *T* = 350 ± 2 K to avoid any condensation and at pressures 0.09–0.25 Torr for 1*H*-1,2,3-triazole and 0.09–0.3 Torr for pyrazole. The concentration of the studied compound was chosen in such way to obtain optical density values between 0.05 and 2.5, conditions for which the linearity of Lambert Beer's law is respected.

### 2.2. Reactions with OH radicals

Kinetic studies were conducted in a photochemical reactor coupled to an FTIR spectrometer and GC/MS. The apparatus and the methodology used in this work were the same as described elsewhere^23,24^ and only a brief review is given here. In short, the reactor, made of Pyrex, is a triple-jacket cell (length 2 m, internal diameter 20 cm) equipped with a multiple reflection system. The temperature of the reaction cell was controlled by the passage of a heating or cooling fluid through a jacket surrounding the Pyrex reaction cell. Platinum resistance temperature sensors were used to provide continuous and simultaneous temperature readings. The pressure inside the cell was measured by (0–1000) Torr MKS Baratron capacitance manometers. The first two outer layers of the chamber delimit a vacuum in order to isolate the whole system from its surroundings. Experiments were carried out over the temperature range 298–357 K and at atmospheric pressure (760 ± 5 Torr).

An Equinox 55 FTIR spectrometer was used to monitor the consumption of the reactants and reference compounds. The spectral resolution was in the range of 2–0.5 cm^−1^ in the spectral domain 600–4000 cm^−1^. 24 UV lamps symmetrically arranged emitting from 300 to 400 nm were used to generate OH by the photolysis of nitrous acid which was produced in a drop-wise by the addition of 10% of sulphuric acid to a solution of 0.2 M of sodium nitrite. A small flow of nitrogen gas was used to carry the generated nitrous acid into the reactor. In this work, benzaldehyde, heptane and *trans*-2-hexenal were used as reference compounds. The reference compounds were chosen in such way that at least one absorption band of the studied compound does not exhibit any interference with those of the chosen references and *vice versa*. Measured amounts of reagents were flushed from calibrated bulbs into the reactor through a stream of ultra-pure air. The reactor was then filled at atmospheric pressure with ultra-pure air. The experimental conditions used for the kinetic study are summarized in Table 1. As can be seen in Table 1, the initial reagents concentrations were chosen to minimize any secondary reactions and to be discernable analytically. It should be noted that, during an experiment, infrared spectra were recorded every 5 minutes. Each spectrum constitutes the average of 20 accumulated spectra with a resolution of 2 cm^−1^. The kinetic monitoring of the reaction medium was conducted until more than 30% consumption of the studied compound and the used reference. In this study the duration of experiments goes from 4 to 6 hours.

**Table 1:** Experimental conditions used for the reactions of 1*H*-1,2,3-triazole and pyrazole with OH radicals in the pyrex simulation chamber coupled to a FTIR spectrometer

	1*H*-1,2,3-Triazole	Pyrazole
Temperature (K)	298–357	298–357
Pressure (Torr)	760 ± 5	760 ± 5
Reference compound	Benzaldehyde, heptane, *trans*-2-hexenal	Benzaldehyde, heptane
Optical path (m)	56–64	56–64
[Triazole or pyrazole] molecules per cm^3^	(2–4) × 10^14^	(7–8) × 10^14^
[Reference] molecules per cm^3^	(4–6) × 10^14^	(5–9) × 10^14^
Spectral range (cm^−1^)	(905–943); (3480–3552)	(3466–3549); (3480–3552)
Spectral range (cm^−1^) (reference)	Benzaldehyde: (2700–2760); (2770–2832)	Benzaldehyde: (2700–2760); (2770–2832)
	Heptane: (1430–1500)	Heptane: (1337–1403); (1430–1500)
	*trans*-2-Hexenal: (2660–2760)	

The reaction medium was also sampled using solid phase microextraction fibers (SPME) mainly polydimethylsiloxane/divinylbenzene fibers (PDMS/DVB) exposed for 1 min into the chamber. The SPME fiber was then introduced into the GC/MS injector operating at 220 °C. The GC/MS analysis were performed in TIC mode (Total Ion Current).

The measurements were performed in purified air provided by Air Liquide (>99.9999%). The reagents: 1*H*-1,2,3-triazole (97%), pyrazole (98%), benzaldehyde (99.5%), *trans*-2-hexenal (98%) and heptane (97%) were provided by Sigma-Aldrich. They were further purified by distillation and by repeated freeze–pump–thaw cycles before use in both the spectroscopic and kinetic studies.

## 3. Results and discussion

### 3.1. UV spectra

The UV absorption cross-section values obtained in the spectral range 200–250 nm, at 350 ± 2 K are presented in SM-1. The reported values represent the average of 10–12 independent measurements at several different pressures. The recorded spectra have been found relatively reproducible with a variation that does not exceed 30%.

**Fig. 1:**
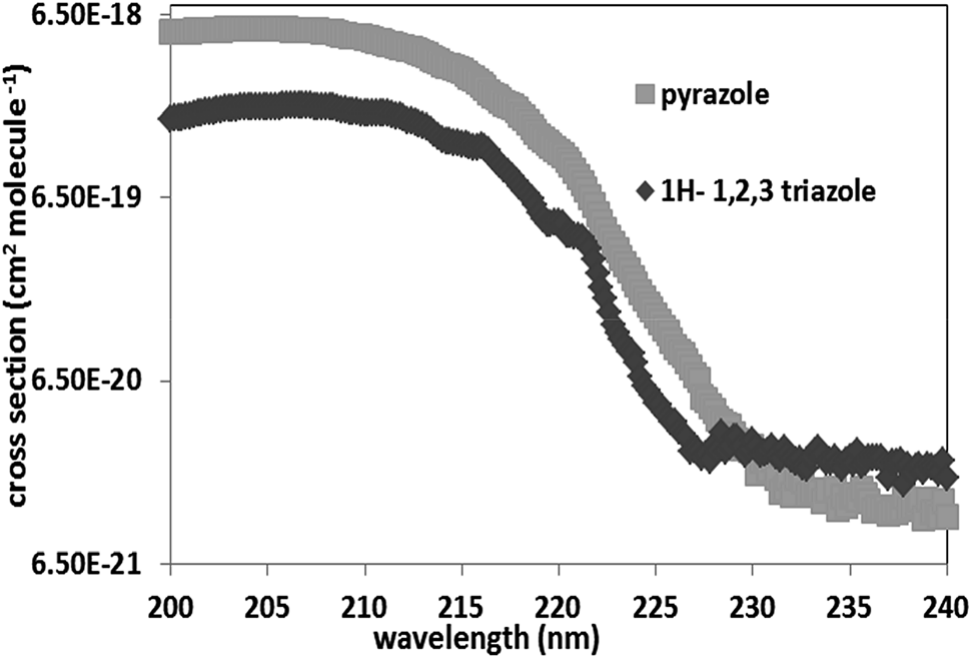
UV absorption spectra of 1*H*-1,2,3-triazole and pyrazole in the range 200–240 nm.

Fig. 1 shows the absorption spectra of 1*H*-1,2,3-triazole and pyrazole. These spectra consist of a broad continuum with a strong absorption band between 200–240 nm. They are very similar in terms of widths, however pyrazole absorbs 3 times more than 1*H*-1,2,3-triazole. The absorption maximum of 1*H*-1,2,3-triazole is localized at 206 nm (*σ*_206, 1*H*-1,2,3-triazole_ = 2.04 × 10^−18^ cm^2^ per molecule) and at 203 nm (*σ*_203, pyrazole_ = 5.44 × 10^−18^ cm^2^ per molecule) for pyrazole. This absorption band is attributed to the π–π* electronic transitions band, characteristic of aromatic compounds. Nevertheless, the n–π* transition was not observed probably due to its low intensity, so that it is completely drowned in the π–π* band.

The addition of a nitrogen atom to the heterocycle exerts an hypsochromic effect of about 4 nm for 1*H*-1,2,3-triazole with respect to the pyrazole and an hypochromic effect since the absorption intensity of 1*H*-1,2,3-triazole is 3 times lower than that of pyrazole.

The absorption of these two compounds beyond 290 nm, spectral range which corresponds to radiation reaching the troposphere, is very low (≤10^−21^ cm^2^ per molecule). Thus, their atmospheric photodissociation processes are negligible.

#### Errors analysis

Between 210–230 nm, uncertainty on UV absorption cross sections are less than 20%. However, uncertainties higher than 30% were obtained for wavelengths >230 nm mainly attributed to the weak optical density of the studied compounds in the studied spectral region (absorbance less than 0.05).

The main error sources are attributed to the low vapor pressure of the studied compounds and their tendency to stick to the wall of the absorption cell, which distorts the pressure measurements and entails an error on the concentration calculation. To minimize this error, we increased the temperature of the cell (353–357 K) so that the uncertainty on the concentration measurements did not exceed 15%.

Other sources of errors in the spectral measurements can result the calibration wavelength, the temperature, the optical length and the absorbance. However, the uncertainty due to these parameters does not exceed 5%.

#### Comparison with the literature

**Fig. 2:**
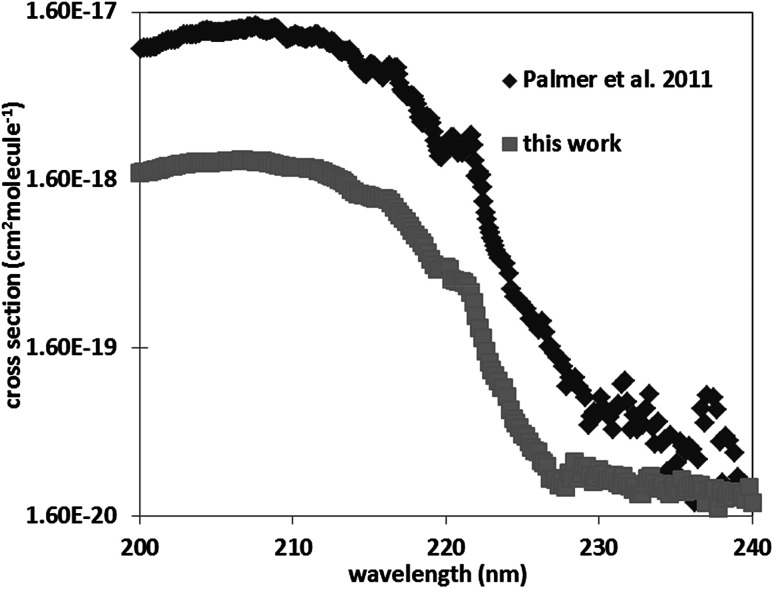
Comparison of the UV spectra of 1*H*-1,2,3-triazole with that of literature.

One study is available in the literature concerning the gas phase UV-Vis absorption spectrum of 1*H*-1,2,3-triazole. Palmer *et al.*^20^ used a synchrotron radiation source to measure the absorption spectrum of 1*H*-1,2,3-triazole in the spectral range 115–245 nm, at 308 K. This spectrum presents the same shape and position of the absorption maximum obtained in our study. However, the absorbance values of 1*H*-1,2,3-triazole obtained in this study is 10 times lower than that reported by Palmer *et al.*^20^ in the spectral range 200–240 nm. It should be noted that the study carried out by Palmer *et al.*^20^ focuse mainly on the band's positions and electronic transitions, with a minor importance to the cross-sections values (Fig. 2).

**Fig. 3:**
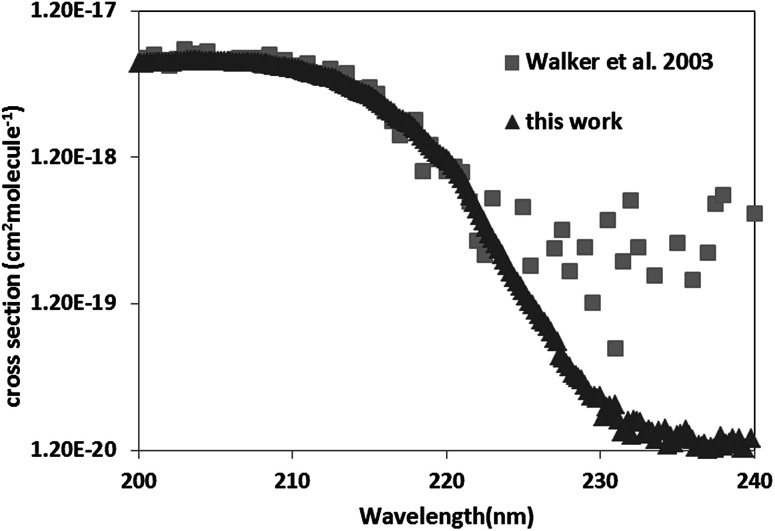
Comparison of the UV spectra of pyrazole with that of literature.

For pyrazole, only one experimental study exists in literature. Walker *et al.*^19^ determined the UV-Vis spectrum of pyrazole between 106 and 250 nm with an increment of 0.05 nm. The obtained spectrum is in a good agreement with that of literature, with cross sections deviations less than 20% in the domain 200–220 nm. Nevertheless, beyond 230 nm, Walker *et al.*^19^ have cross sections 2 to 10 times higher than ours. Beyond 220 nm the cross sections of Walker *et al.*^19^ oscillates around 5 × 10^−19^ cm^2^ per molecule including negative values and are very noisy explaining our discrepancies (Fig. 3).

### 3.2. Reaction with OH radicals

The OH reactivity of 1*H*-1,2,3-triazole and pyrazole was investigated using the relative mode in the temperature domain 298–357 K, at atmospheric pressure. Three different references were used: benzaldehyde, *n*-heptane and *trans*-2-hexenal. Their choice was based on three essential criteria: (i) the reference compound have a well-documented kinetic rate constant of their homogeneous OH-oxidation; (ii) the OH-reactivity of the reference compounds are of the same order of magnitude as that of the analyte; (iii) the FTIR spectrum of the reference compounds have at least one absorption band that does not interfere with the analyte spectrum and *vice versa*.

The rate coefficients of the reaction of OH radicals with the reference compounds used are (in cm^3^ per molecule per s):

1 *k*_(OH+benzaldehyde)_ (*T*) = 5.33 × 10^−12^ exp((2020 ± 710)/*RT*) (**ref. 25**)

2 *k*_(OH+*trans*-2-hexenal)_ (298 K) = (4.44 ± 0.94) × 10^−11^

3 *k*_(OH+*n*-heptane)_ (298 K) = (6.68 ± 0.48) × 10^−12^

The compound and the reference are simultaneously subjected to oxidation by OH radicals. The reactions taking place in the reactor are:Analyte + OH → products, *k*_OH_Analyte → products, *k*_p_Reference + OH → products, *k*_ref_
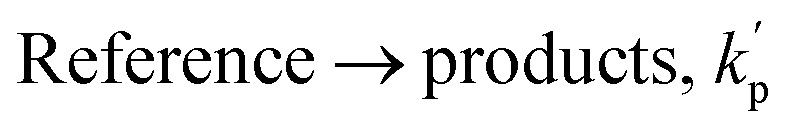


Secondary processes such as wall loss and photolysis under UV radiation can occur. In order to evaluate these losses, experiments were conducted in the absence of UV radiation and/or HONO. These experiments showed that the photolysis of these compounds and their reaction with HONO are negligible. However, wall losses were (2.50 ± 0.50) × 10^−5^ s^−1^ for 1*H*-1,2,3-triazole and (3.50 ± 0.50) × 10^−5^ s^−1^ for pyrazole.

To take into account to the wall loss, the following relation was used to determine the rate constants of the reaction of OH radicals with 1*H*-1,2,3-triazole and pyrazole:II
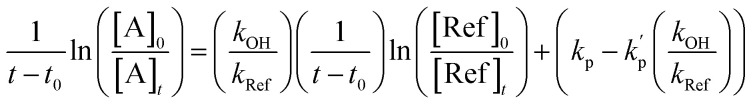
where [A]_0_ and [Ref]_0_ are the initial concentrations of 1,2,3-triazole and pyrazole and the reference at time *t* = 0, [A]_*t*_ and [Ref]_*t*_ are the concentrations of 1,2,3-triazole and pyrazole and the reference at time *t*, and *R* is the ratio of the OH-oxidation rate constants of the analyte (A) and the reference (Ref) (*R* = *k*_OH_/*k*_Ref_). *k*_p_ and 
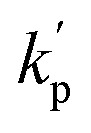
 are the sum of the rate coefficients due to the loss of the nitrogenous heterocyclic compounds and the references, respectively, by secondary reactions such as wall losses and photolysis.

**Fig. 4:**
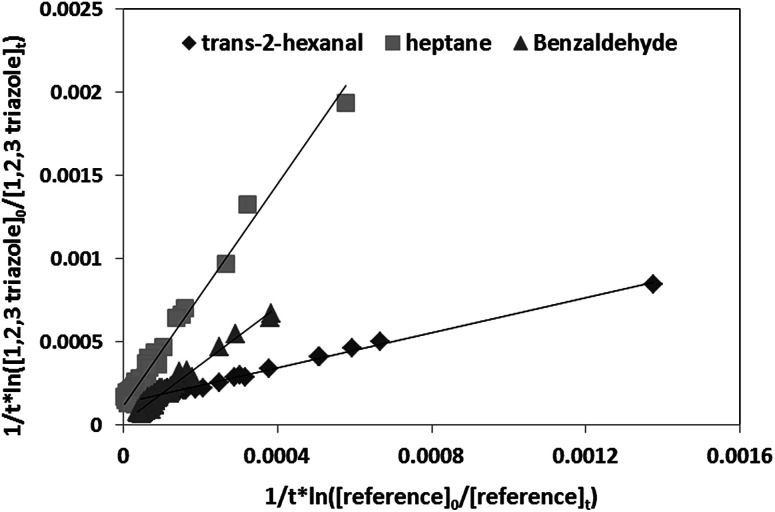
Relative kinetics of the reaction of the OH radicals with 1*H*-1,2,3-triazole for different references obtained by FTIR at 298 K.

**Fig. 5:**
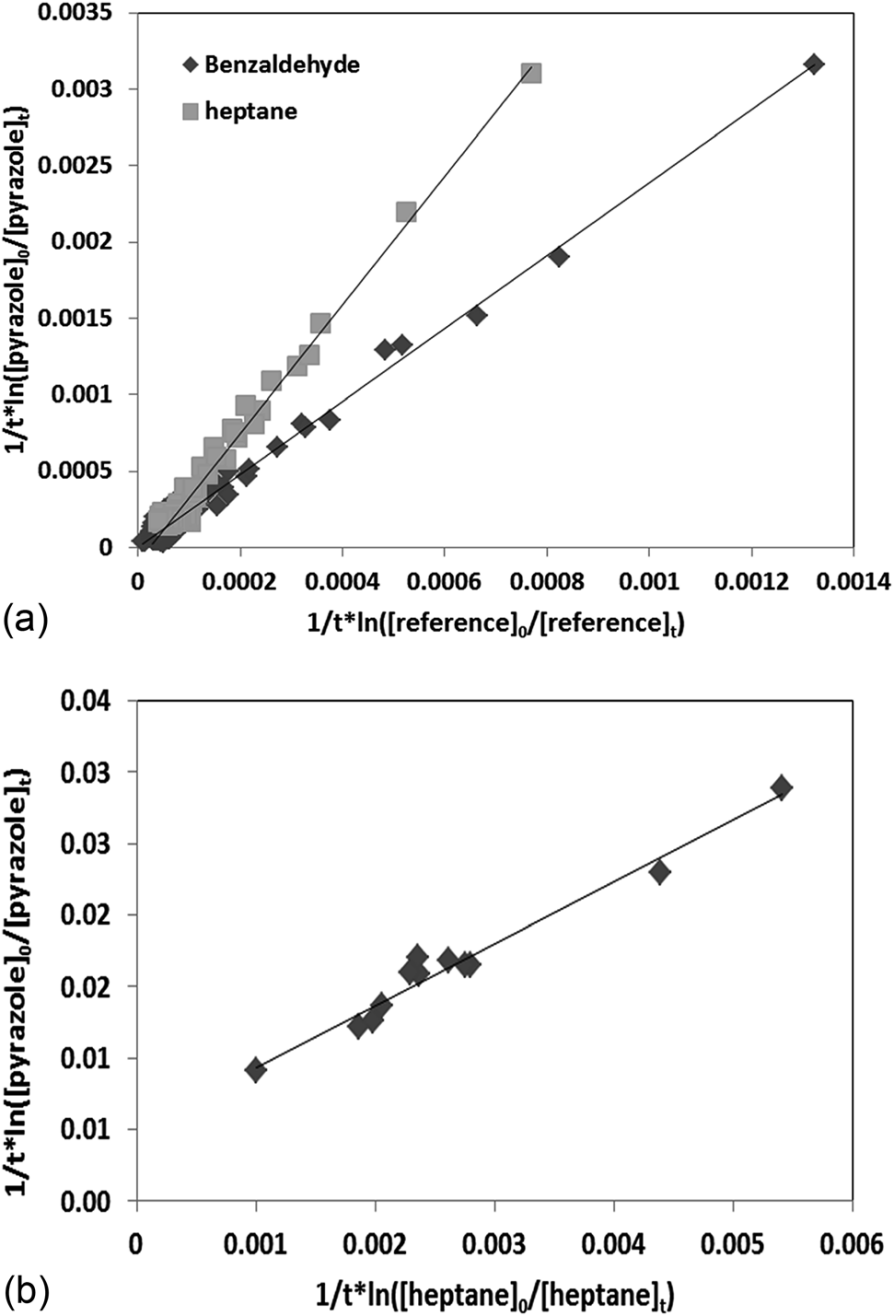
(a) Relative kinetics of the reaction of the OH radicals with pyrazole for different references obtained by FTIR at 298, (b) relative kinetics of the reaction of the OH radicals with pyrazole using heptane as a reference obtained by SPME-GC/MS at 298 K.

According to eqn (II), the plot of 
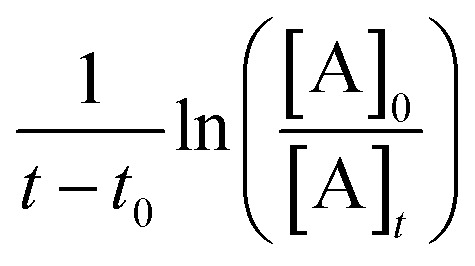
 as a function of 
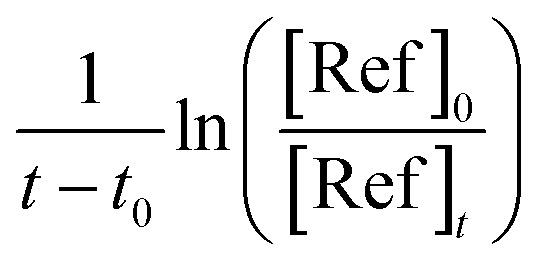
 is a straight line whose slope is equal to *R* = *k*_OH_/*k*_Ref_. Knowing the value of *k*_Ref_ from literature, we can deduce *k*_OH_ for the studied compounds. Fig. 4 and 5(a) and (b) show an example of this plot for 1*H*-1,2,3-triazole and pyrazole at room temperature obtained by FTIR and GC/MS with different references. Good linearity is observed with a correlation coefficient greater than 94%.

The rate constants of the reaction of 1*H*-1,2,3-triazole and pyrazole with OH radicals obtained using different references at ambient temperature are very close with a difference ≤15%. Given the low vapor pressure of these compounds, temperature studies were carried out between 298 and 357 K, using benzaldehyde as a reference. The rate coefficients obtained at different temperatures by FTIR (1*H*-1,2,3-triazole and pyrazole) and GC/MS (pyrazole) are summarized in Table 2. Each experiment was performed three times under the same experimental conditions. The relative error on the slope corresponds to one standard deviation.

**Table 2:** Rate constants of OH-oxidation of 1,2,3-triazole and pyrazole at different temperatures obtained by FTIR and SPME-GC/MS^b^^,^^c^

References	*T* (K)	1*H*-1,2,3-Triazole		Pyrazole	
		*R* = *k*_OH_/*k*_Ref_	*k*_1*H*-1,2,3-triazole+OH_ (10^−11^ cm^3^ per molecule per s)	*R* = *k*_OH_/*k*_Ref_	*k*_pyrazole+OH_ (10^−11^ cm^3^ per molecule per s)
Benzaldehyde	298	1.66 ± 0.14	2.00 ± 0.60	2.43 ± 0.10	2.93 ± 0.84
	313	1.61 ± 0.10	1.86 ± 0.51	2.36 ± 0.10	2.73 ± 0.78
	333	1.46 ± 0.10	1.61 ± 0.41	2.13 ± 0.13	2.36 ± 0.62
	357	1.40 ± 0.10	1.47 ± 0.35	2.14 ± 0.10	2.25 ± 0.54
Heptane	298	3.31 ± 0.02	2.22 ± 0.16	4.27 ± 0.10	2.91 ± 0.20
				4.38 ± 0.11^a^	2.98 ± 0.22^a^
*trans*-2-Hexenal	298	0.51 ± 0.01	2.26 ± 0.48	—	—

a Results obtained in SPME-GC/MS.

b Uncertainty on *R* is 1*σ*.

c Uncertainty on *k*_1*H*-1,2,3-triazole+OH_ and *k*_pyrazole+OH_ calculated with error propagation method.

**Fig. 6:**
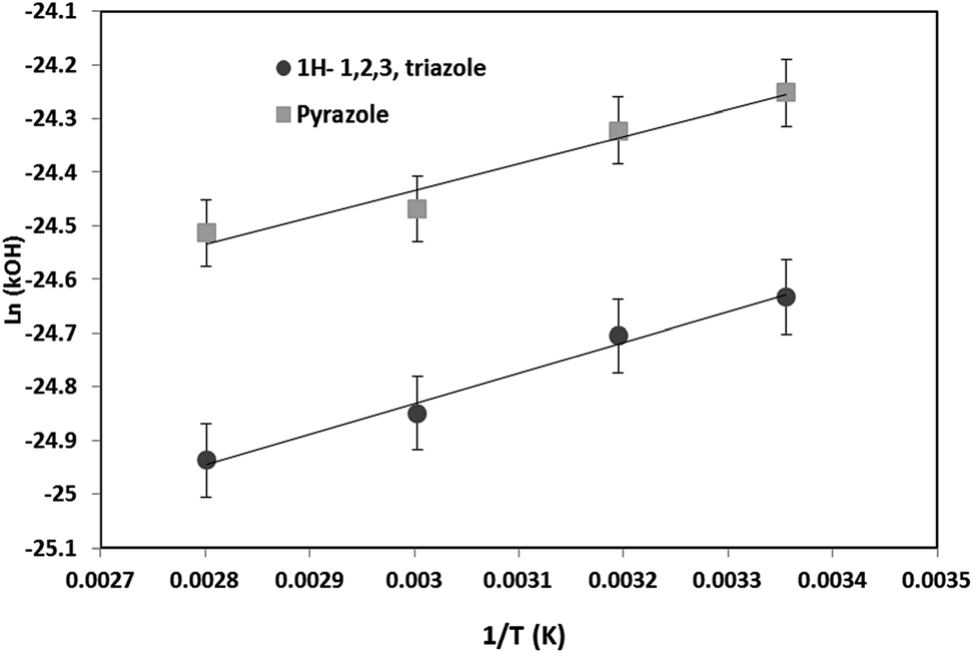
Arrhenius plot for the reaction of 1*H*-1,2,3-triazole and pyrazole with OH radicals.

The Arrhenius diagram was then determined by plotting ln(*k*_OH_) as a function of 1/*T* (Fig. 6). The Arrhenius equations for the OH oxidation of 1*H*-1,2,3-triazole and pyrazole were as follows (in cm^3^ per molecule per s):*k*_OH+1*H*-1,2,3-triazole_ (*T*) = (2.93 ± 0.20) × 10^−12^ exp((570 ± 43)/*T*)*k*_OH+pyrazole_ (*T*) = (5.42 ± 0.60) × 10^−12^ exp((499 ± 74)/*T*)

Uncertainties on the Arrhenius parameters (activation energy *E*_a_ and pre-exponential factor *A*) were determined by the least square minimization method.

The obtained kinetic data show that the OH-oxidation of 1*H*-1,2,3-triazole and that of pyrazole exhibit a low negative temperature dependence. The rate constants decrease by about 26% for 1*H*-1,2,3-triazole and 28% for pyrazole when the temperature goes from 298 K to 357 K.

#### Error analysis

The error on rate coefficients has two components: (i) random error due to unpredictable variations during the experience. The random error is reduced by increasing the number of experiments and therefore its contribution can be considered negligible; (ii) systematic error caused by a perfectly identified and quantifiable effect of an experiment. This error component remains constant or varies predictably. In our experiments the evaluation of these two errors is very complex given the multiplicity of devices used and the diverse measured parameters. Overall uncertainty on rate constants was determined by the error propagation method, using the relationship (III)):III
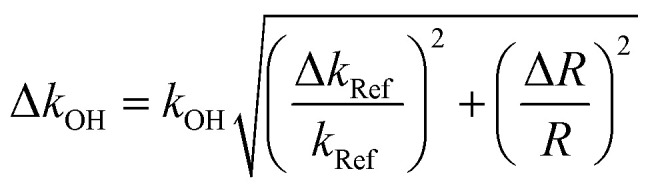


Considering (III)) the main error sources in this study are:

• The uncertainty on the rate constant of the reference given by the literature *k*_Ref_ varies between 5 and 20%.

• The experimental error corresponds to the uncertainty on the determination of the slope 
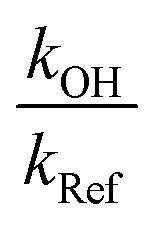
 which generally depends on the integration accuracy of the IR bands and chromatographic peaks. This error is minimized by the repetition of several experiments, and the kinetic tracking of IR bands that do not interfere with parasitic peaks. Indeed, this error is estimated between 5% to 15%.

We can note that the kinetic measurements performed with different references and analytical techniques (IR and GC/MS) were reproducible. An excellent agreement between the different determinations was observed with a difference that does not exceed 8%.

#### Temperature effect and reaction mechanism

The OH-oxidation of 1,2,3-triazole and pyrazole is not sensitive to temperature since the obtained rate coefficients showed a negative temperature dependence. The temperature coefficients for both compounds are very close indicating that probably both compounds undergo the same reaction mechanism during the attack by OH radicals. This reaction can be carried out initially without an activation energy barrier. This trend shows that processes involved in this reaction are a succession of elementary steps involving unstable intermediate complexes.

In a study carried out by Samuni *et al.*^28^ on the reaction of OH radicals with pyrazole in aqueous phase, it was proved that this reaction mainly occurs by adding the OH radical to the adjacent carbon of the nitrogen atom. In addition, Dillon *et al.*^17^ observed that the gas phase oxidation of pyrrole, a compound having a chemical structure similar to the compounds studied in this work, by OH radicals follows the same trend concerning the variation of the rate constant with the temperature: *k*_OH_ slightly varies with temperature exhibiting a negative temperature coefficient. These authors also performed a theoretical study to elucidate the mechanism of the reaction between pyrrole and OH radicals. The results showed that the reaction is preferably carried out by adding the OH radical to the pyrrole ring (C_4_H_5_N). This process leads to the formation of a pre-reactive complex where the OH radical forms a hydrogen bond with the aromatic π system, slightly excentric on the carbon side of C_4_H_5_N. The most favorable addition concerns the carbons adjacent to the nitrogen atom. By analogy to the mechanism established for the OH-oxidation of pyrazole in the aqueous phase^28^ and those of pyrrole in the gas phase,^17^ we can suppose the OH-reaction of 1*H*-1,2,3-triazole and pyrazole follows the same mechanism as that described above.

#### Comparison with the literature

To our knowledge, this work is the first kinetic determination of the reaction of OH radicals with pyrazole and 1*H*-1,2,3-triazole. However, these kinetic data can be compared to the OH-oxidation kinetic constants of other heterocyclic aromatic compounds with 5 atoms (Table 3).

**Table 3:** Comparison of the rate constants of the reactions of OH radicals with 1*H*-1,2,3-triazole and pyrazole with that of homologous compounds with five atoms from literature

Compounds	*T* (K)	*k* (cm^3^ per molecule per s)	Techniques^a^	References
Pyrrole	298 ± 2	(1.20 ± 0.04) × 10^−10^	RM^b^	16
	298 ± 2	(1.05 ± 0.06) × 10^−10^	FP-FR	17
	298 ± 2	(1.28 ± 0.10) × 10^−10^	PL-FIL	18
1,2,3-Triazole	298 ± 2	(2.00 ± 0.60) × 10^−11^	RM^c^	This work
	298 ± 2	(2.22 ± 0.20) × 10^−11^	RM^d^	This work
	298 ± 2	(2.26 ± 0.48) × 10^−11^	RM^e^	This work
Pyrazole	298 ± 2	(2.93 ± 0.84) × 10^−11^	RM^c^	This work
	298 ± 2	(2.91 ± 0.2) × 10^−11^	RM^d^	This work
	298 ± 2	(2.98 ± 0.22) × 10^−11^	RM^e^	This work
Thiophene	298	(9.6 ± 0.3) × 10^−12^	RM^f^	31
Furan	298	(4.1 ± 0.3) × 10^−11^	RM^f^	32

a RM: relative method; RE-MS: flow reactor mass spectrometry; FP-FR: flash photolysis resonance fluorescence; PL-FIL: laser-induced fluorescence laser photolysis.

b Reference: propene.

c Reference benzaldehyde, analysis by FTIR.

d Reference heptane, analysis by FTIR.

e Reference heptane, analysis in GC/MS.

f Reference: *n*-hexane.

The data presented in this table highlight the following points:

• The rate constants of the heterocyclic compounds with five atoms are of the same order of magnitude.

• Pyrrole is the most reactive towards OH radicals with a ten times higher rate constant than 1*H*-1,2,3-triazole and pyrazole. Thus, replacing a carbon atom by a nitrogen in 5 atoms aromatic ring induces a deactivating effect on the reaction: *k*(OH + pyrrole) ≫ *k*(OH + pyrazole) > *k*(OH + 1*H*-1,2,3-triazole).

According to the SAR method^29^ which estimates OH reaction rate coefficient for gaseous phase of compounds based on molecular structure, a difference of 15% is observed between the experimental value and the estimated one for the OH-reaction of pyrazole. However, for 1*H*-1,2,3-triazole the difference is very large, the experimental value being 200 times higher than the estimated value. This discrepancy can be explained by the fact that, for this compound the SAR procedure uses an addition factor of 0.1 × 10^−12^. This value is based on the kinetic of the reaction between the OH radicals and 1,2,3 triazine,^30^ a compound whose structure is not similar to that of 1*H*-1,2,3-triazole. Consequently, the experimental value determined in this work for the rate coefficient of OH-reaction with 1*H*-1,2,3-triazole could be used as an addition factor to estimate the rate constants between OH radicals and chemical compounds having a chemical structure close to that of 1*H*-1,2,3-triazole.

## 4. Atmospheric implications

The spectroscopic and the kinetic data obtained in this work were used to estimate the tropospheric lifetimes of 1*H*-1,2,3-triazole and pyrazole with respect to the different processes that lead to their atmospheric elimination. These processes are photolysis and their reactions with the main atmospheric oxidants (OH, O_3_). In the case of reactions with an atmospheric oxidant X, the lifetime of a compound is estimated according to the equation:

IV *τ* = 1/*k*_x_[X]

*k*_x_ is the rate constant of the biomolecular reaction and [X] is the average concentration of the atmospheric oxidant.

The lifetime values *τ* of 1*H*-1,2,3-triazole and pyrazole with respect to OH radicals are 14 h and 9 h respectively. A 24 hours average concentration of OH radicals in the atmosphere equal to 1 × 10^6^ molecule per cm^3^ was used in the calculation.^32^

The atmospheric lifetimes of these compounds are relatively short (few hours) indicating that these compounds are non-persistent and can undergo a fast-photochemical transformation close to their emission source contributing therefore to the photochemical pollution mainly at the local scale.

## 5. Conclusions

In this work, the UV absorption spectra and temperature dependent kinetic data of the reaction of two five-membered nitrogen heterocycles (1*H*-1,2,3-triazole and pyrazole) with respect to OH radicals were determined. Spectroscopic studies were performed in the spectral domain 200–300 nm. The absorption spectra of 1*H*-1,2,3-triazole and pyrazole showed a very low absorption capacity beyond 240 nm, so their atmospheric photodissociation is a negligible degradation process. The cross-section values obtained in the spectral range 210–230 nm have thus been determined with a good accuracy and can be used for the quantification of these compounds in both the laboratory and the atmosphere. Moreover the reactivity of these species with OH radicals was performed in an atmospheric simulation chamber coupled to an FTIR spectrometer and to a GC/MS using the relative mode. Kinetic data showed that the OH-oxidation of 1*H*-1,2,3-triazole and pyrazole exhibit a negative temperature dependence over the temperature range 298–357 K and the addition of a nitrogen atom (substituting a carbon atom) in the aromatic ring exerts a deactivating effect on the reaction with OH radicals. Indeed, the kinetic data obtained in this work will contribute to improve the SAR method used to estimate the rate coefficient of the gas phase reaction of OH radicals with five-membered nitrogen heterocycle compounds. Additionally, it was found that these compounds are mainly eliminated from the atmosphere following their reaction with OH radicals with lifetimes of order of few hours. Thus, these compounds are not persistent in the troposphere and can induce photochemical pollution at the local scale.

Finally, it is noteworthy to mention that information regarding the atmospheric oxidation mechanisms of these species are still required to better evaluate their atmospheric impacts. Quantum chemistry calculations concerning the OH-oxidation kinetic of these compounds are yet necessary to explore in depth the reactions mechanisms and to fully explain the present experimental findings regarding their structural effect on their reactivity.

## Conflicts of interest

There are no conflicts to declare.

## Supplementary Material

RA-009-C9RA04235K-s001
