# Supplementary material for: Gas-phase UV absorption spectra and OH-oxidation kinetics of 1H-1,2,3-triazole and pyrazole
Source: RSC Adv. 2019 Aug 30;9(47):27361–8. doi: 10.1039/c9ra04235k (PMC9070650; doi:10.1039/c9ra04235k)
Supplement: RA-009-C9RA04235K-s001 [file RA-009-C9RA04235K-s001.pdf]

Electronic Supplementary Material (ESI) for RSC Advances.
This journal is © The Royal Society of Chemistry 2019

SM-1: UV-visible absorption cross sections σ (cm^2 $molecule^{-1}$) of 1H-1.2.3 triazole and pyrazole.

λ (nm)	σ (cm^2 $molecule^{-1}$)	
	1H- 1.2.3 triazole	Pyrazole
**200**	(1.75±0.12) 10^{-18}	(5.23±0.86) 10^{-18}
**201**	(1.83±0.10) 10^{-18}	(5.30±0.89) 10^{-18}
**202**	(1.93±0.12) 10^{-18}	5.34±0.89) 10^{-18}
**203**	(1.99±0.12) 10^{-18}	(5.44±0.94) 10^{-18}
**204**	(2.05±0.13) 10^{-18}	(5.48±0.94) 10^{-18}
**205**	(2.05±0.13) 10^{-18}	5.44±0.93) 10^{-18}
**206**	(2.09±0.14) 10^{-18}	(5.42±0.90) 10^{-18}
**207**	(2.09±0.19) 10^{-18}	(5.33±0.88) 10^{-18}
**208**	(2.06±0.19) 10^{-18}	(5.27±0.87) 10^{-18}
**209**	(1.970.18) 10^{-18}	(5.04±0.78) 10^{-18}
**210**	(1.90±0.17) 10^{-18}	(4.88±0.76) 10^{-18}
**211**	(1.88±0.19) 10^{-18}	(4.58±0.74) 10^{-18}
**212**	(1.78±0.17) 10^{-18}	(4.29±0.66) 10^{-18}
**213**	(1.61±0.14) 10^{-18}	(4.01±0.58) 10^{-18}
**214**	(1.36±0.12) 10^{-18}	(3.51±0.52) 10^{-18}
**215**	(1.28±0.11) 10^{-18}	(3.19±0.41) 10^{-18}
**216**	(1.24±0.1) 10^{-18}	(2.68±0.40) 10^{-18}
**217**	(9.82±0.9) 10^{-19}	2.21±0.28) 10^{-18}
**218**	(7.54±0.68) 10^{-19}	(1.87±0.34) 10^{-18}
**219**	(5.38±0.51) 10^{-19}	(1.42±0.18) 10^{-18}
**220**	(4.82±0.51) 10^{-19}	(1.17±0.15) 10^{-18}
**221**	(3.99±0.87) 10^{-19}	(8.52±012) 10^{-19}
**222**	(2.49±0.67) 10^{-19}	(5.32±0.82) 10^{-19}
**223**	(1.18±0.30) 10^{-19}	(3.33±0.51) 10^{-19}

**224**	(8.17±2.00) 10^{-20}	(2.18±0.35) 10^{-19}
**225**	(4.88±1.23) 10^{-20}	(1.46±0.27) 10^{-19}
**226**	(3.85±0.85) 10^{-20}	1.00±0.26) 10^{-19}
**227**	(2.69±0.32) 10^{-20}	(6.97±0.14) 10^{-20}
**228**	(2.88±0.63) 10^{-20}	(4.43±0.54) 10^{-20}
**229**	(3.17±0.39) 10^{-20}	3.30±0.34) 10^{-20}
**230**	(3.03±0.51) 10^{-20}	(2.81±0.42) 10^{-20}
**231**	(2.81±0.80) 10^{-20}	(2.42±0.26) 10^{-20}
**232**	(2.42±0.71) 10^{-20}	(1.52±0.43) 10^{-20}
**233**	(2.46±0.80) 10^{-20}	(1.59±0.57) 10^{-20}
**234**	(2.33±0.70) 10^{-20}	(1.66±0.57) 10^{-20}
**235**	(2.45±0.17) 10^{-20}	(1.64±0.29) 10^{-20}
**236**	(2.44±0.22) 10^{-20}	(1.67±0.35) 10^{-20}
**237**	(1.90±0.45) 10^{-20}	(1.14±0.27) 10^{-20}
**238**	(2.25±0.70) 10^{-20}	(1.27±0.27) 10^{-20}
**239**	(2.23±0.20) 10^{-20}	(1.70±0.37) 10^{-20}
**240**	(1.90±0.49) 10^{-20}	(1.16±0.30) 10^{-20}
**241**	(2.21±0.65) 10^{-20}	(9.67±3.41) 10^{-20}
**242**	(2.02±0.52) 10^{-20}	(1.31±0.19) 10^{-20}
**243**	(2.12±0.43) 10^{-20}	(1.07±0.18) 10^{-20}
**244**	(2.21±0.40) 10^{-20}	(9.75±1.35) 10^{-21}
**245**	(1.89±0.57) 10^{-20}	(8.87±3.08) 10^{-21}
**246**	(2.02±0.55) 10^{-20}	(1.13±0.34) 10^{-20}
**247**	(1.96±0.25) 10^{-20}	(9.55±2.51) 10^{-21}
**248**	(1.99±0.68) 10^{-20}	(9.52±3.50) 10^{-21}
**249**	(2.18±0.62) 10^{-20}	(9.26±1.60) 10^{-21}
**250**	(2.05±0.48) 10^{-20}	(1.29±0.23) 10^{-20}
